# Pan-genome landscape of the JAZ family in pepper (*Capsicum annuum* L.): core-variable architecture and hormone crosstalk in leaves

**DOI:** 10.3389/fpls.2026.1798486

**Published:** 2026-04-13

**Authors:** Huaming Zhong, Xiaolin Song, Qingbin Chen, Lingyi Wang, Jinhang Guo, Bo Wang, Zhuoye Xie, Tianqi Niu, Jiadong Shi, Meng Tang, Jie Yang

**Affiliations:** College of Biology and Food, Shangqiu Normal University, Shangqiu, Henan, China

**Keywords:** adaptive evolution, *Capsicum annuum* L., JAZ gene family, pan-genome, phytohormone crosstalk, presence/absence variation

## Abstract

The JASMONATE ZIM-DOMAIN (JAZ) proteins are master repressors of jasmonate (JA) signaling and central hubs for phytohormone crosstalk. While JAZ family evolution has been studied in model plants, their pan-genome dynamics and transcriptional specialization in pepper (*Capsicum annuum* L.), a globally important Solanaceous crop, remain unexplored. In this study, we conducted a comprehensive pan-genome analysis of the JAZ family across 20 diverse pepper accessions. We identified 12 *CaJAZ* members exhibiting a dichotomous evolutionary pattern: core genes (*CaJAZ2*/*3*/*4*/*5*/*7*) under strong purifying selection versus dispensable genes (*CaJAZ6*/*9*) showing lineage-specific structural variations. Notably, *CaJAZ9* underwent extensive tandem duplication (up to 5 copies in specific landraces), while *CaJAZ6* exhibited adaptive loss in cultivated varieties, suggesting diversifying selection. Promoter analysis revealed prevalent light-responsive elements (Box 4/G-box) and hormone-specific motifs (ABRE, CGTCA). Crucially, structural parsing of the Jas motif unmasked a highly stratified receptor-interacting matrix. We discovered that the structural truncation of the universally conserved Pro-Tyr (P-Y) anchor in CaJAZ6 perfectly uncouples it from MeJA-induced degradation, rendering it “transcriptionally deaf” and acting as a recalcitrant molecular brake. Transcriptionally, the family displayed distinct temporal modules: rapid transient activation by MeJA (peak at 1 h), sustained biphasic responses to ABA (secondary peak at 12 h), and delayed induction by SA (12–24 h). A functional “division of labor” was observed, with *CaJAZ3*/*4*/*9* acting as JA-specific sentinels and *CaJAZ1*/*2*/*5* serving as multi-hormone integrators. This study provides the first pan-genome atlas of the pepper JAZ family, revealing that structural variation (PAV/CNV/motif anomalies) and promoter architecture underlie adaptive diversification. The identification of core versus variable haplotypes offers precise genetic targets for breeding climate-resilient pepper varieties with optimized growth-defense trade-offs.

## 1 Introduction

As sessile organisms, plants have evolved highly adaptable signaling networks to coordinate growth and development while concurrently dealing with fluctuating environmental cues and biotic attacks. Among the phytohormone signaling pathways, Jasmonates (JAs) act as a central cellular hub, coordinating the critical trade-off between resource-intensive defense responses and vegetative growth (Browse, 2009; Major et al., 2017). The molecular mechanism of JA signaling is centered on the JASMONATE ZIM-DOMAIN (JAZ) proteins, which act as transcriptional repressors. The discovery of JAZ proteins was a milestone event in plant biology, as it identified them as the missing link between the SCF (COI1) E3 ubiquitin ligase complex and the MYC transcription factors (Chini et al., 2007; Thines et al., 2007). In the resting state, JAZ proteins repress gene expression by recruiting the co-repressor TOPLESS (TPL) through the adaptor protein NINJA (Pauwels et al., 2010). Upon the perception of stress or developmental signals, the accumulation of the bioactive lipid jasmonoyl-L-isoleucine (JA-Ile) promotes the formation of a co-receptor complex between the F-box protein COI1 and JAZs. This interaction initiates the ubiquitination and 26S proteasome-mediated degradation of JAZ repressors, thus releasing downstream transcription factors, such as MYC2, MYC3, and MYC4, to activate specific transcriptional programs (Sheard et al., 2010; Howe and Yoshida, 2019).

Structurally, JAZ proteins are characterized by two highly conserved domains: the N-terminal TIFY domain (also referred to as ZIM), which facilitates homo- and heterodimerization and interaction with NINJA, and the C-terminal Jas domain, which is crucial for JA-dependent interaction with COI1 and binding to transcription factors (Bai et al., 2011; Garrido-Bigotes et al., 2019). Evolutionary genomic analyses indicate that the *JAZ* gene family has experienced substantial expansion during the shift from aquatic algae to land plants, driven primarily by whole-genome duplication (WGD) and segmental duplication events (Bai et al., 2011). This expansion has promoted functional diversification and neofunctionalization, enabling JAZ members to form complex regulatory modules. Consequently, JAZ proteins not only regulate defense against herbivores and necrotrophic pathogens but also modulate diverse abiotic stress responses, such as cold, salt, and drought tolerance (Hu et al., 2013; Li et al., 2014; Du et al., 2021; Zhao et al., 2024). Furthermore, they play a pivotal role in developmental processes such as root architecture remodeling (Raya-González et al., 2012), anthocyanin accumulation (Qi et al., 2011), leaf senescence, and fruit ripening (Yu et al., 2016; Song et al., 2022). In the Solanaceae family, functional characterizations in tomato (*Solanum lycopersicum* L.) and eggplant (*Solanum melongena* L.) have highlighted the distinct roles of specific JAZ isoforms in regulating immunity and chlorophyll metabolism (Sun et al., 2021; Li et al., 2024), indicating species-specific adaptations.

Pepper (*Capsicum annuum* L.) is globally one of the most economically significant vegetable and spice crops, valued for its pungency, nutrition, and diverse fruit phenotypes. While the release of the pepper reference genome (Qin et al., 2014) has facilitated the identification of numerous gene families, a single reference genome fails to capture the extensive genetic diversity and structural variations present within the genus. The “pan-genome” concept has recently emerged as a transformative approach to decipher the complete genomic landscape of a species, distinguishing between “core” genes present in all individuals and “dispensable” genes that show presence/absence variations (PAVs) (Ou et al., 2018). These dispensable genes are often enriched for functions related to environmental adaptation and stress resilience (Shi et al., 2023). Although *JAZ* gene families have been systematically characterized in major crops like wheat (*Triticum aestivum* L.) (Zhai et al., 2024), cotton (*Gossypium hirsutum*) (Li et al., 2017), and maize (*Zea mays* L.) (Han and Luthe, 2021), a comprehensive pan-genome-based analysis of the *JAZ* family in *Capsicum* is lacking. Investigating the PAVs and evolutionary dynamics of *JAZ* genes in pepper is crucial for understanding how this crop adapts to diverse environments and how it regulates specific traits like fruit development.

To address these gaps, we performed a comprehensive pan-genome-based analysis of the *JAZ* gene family across 20 cultivated and wild pepper genomes. We systematically characterized the phylogenetic relationships, gene structures, and conserved motifs of the *CaJAZ* family. Crucially, we investigated the intraspecific diversity through presence/absence variation (PAV) analysis and assessed evolutionary selection pressures via Ka/Ks analysis. Additionally, promoter cis-element profiling was predicted to elucidate potential regulatory complexes. Furthermore, we dissected the structural divergence of the Jas motif to predict receptor binding affinities. Finally, utilizing high-resolution transcriptomic data, we examined the spatiotemporal expression patterns of *CaJAZ* genes under four key hormonal treatments—abscisic acid (ABA), ethylene (ET), methyl jasmonate (MeJA), and salicylic acid (SA)—across multiple time points. This study provides the first pan-genomic insight into the *JAZ* family in pepper, offering valuable candidate genes and theoretical foundations for molecular breeding aimed at enhancing stress resistance and agronomic performance.

## 2 Materials and methods

### 2.1 Data acquisition

The JAZ protein sequences for Arabidopsis (*Arabidopsis thaliana*) and rice (*Oryza sativa*) were retrieved from the TAIR database (https://www.arabidopsis.org/) and the Gramene database (https://www.gramene.org/), accessed on 14 July 2025. The Hidden Markov Models (HMMs) of the TIFY (PF06200) and Jas (PF09425) domains were downloaded from the InterPro database (https://www.ebi.ac.uk/interpro/, accessed on 14 July 2025). The genomic assemblies and annotations of rice and Arabidopsis were acquired from Phytozome (https://phytozome-next.jgi.doe.gov/, accessed on 14 July 2025). The pan-genome assemblies and genomic annotations of pepper were retrieved from http://www.bioinformaticslab.cn/files/genomes/(accessed on 14 July 2025). The information of pepper genomes used in this study was presented in the **Supplementary Material Table 1**. The protein databases of pepper were extracted using TBtools v2.31 (Chen et al., 2023) for subsequent analysis.

### 2.2 Identification of JAZ family members

A HMM-based screening was employed to identify JAZ homologs. TIFY and Jas domains were individually searched using HMMER 3.3.2, with a threshold of E-value < 1e-5. The protein sequences identified from both searches were combined, and redundant sequences (sequences that were identical due to multiple matches) were eliminated. The resulting hits were then merged and submitted to SMART (http://smart.embl-heidelberg.de/) (Letunic and Bork, 2025) and NCBI’s Conserved Domain Database (CDD) (Wang et al., 2023) to verify the presence of TIFY and Jas domains. The information of all identified pepper *JAZ* genes was presented in the **Supplementary Material Table 2**.

### 2.3 Presence/absence variation and nomenclature for gene family members

For each genome, the longest transcript per gene was retained using AGAT, coding sequences were extracted with TransDecoder v5.7.1 (https://github.com/TransDecoder/TransDecoder), and gene models were standardized and converted into BED format using JCVI v1.5.3 (Tang et al., 2024). A representative accession (Ac1979) was used as the initial template for pangenome construction. Genomes were iteratively aligned to the template with JCVI compara.catalog ortholog (--cscore 0.8), and genes lacking syntenic anchors were incrementally appended, enabling progressive expansion of the reference. Redundancy was removed by high-stringency self-comparison of the expanded pangenome (--cscore 0.99), followed by coordinate- and sequence-based filtering. The resulting non-redundant CDS and BED sets were aligned back to each genome using JCVI to obtain comprehensive synteny maps. High-confidence orthologs were defined as the intersection of JCVI syntenic anchors and one-to-one best matches identified from LAST alignments via custom scripts. Presence–absence patterns were summarized by aggregating genes sharing identical pangenome IDs to generate the final genome-by-gene matrix. Heatmap was used to depict the presence/absence of each *JAZ* in the 20 accessions using Rscript v4.0.3 and the ComplexHeatmap package v2.26 (Gu, 2022).

According to the PAV analysis, CA272 was selected as the reference genome for naming the *JAZ* genes. The *JAZ* genes were designated in ascending order of their physical positions, beginning with chromosome 1, based on their coordinates in the CA272 reference, namely *CaJAZ1* to *CaJAZ12*.

### 2.4 Phylogenetic analysis of CaJAZ gene family

To elucidate the evolutionary relationships and diversification patterns of *CaJAZ* genes across pepper germplasms, a comprehensive phylogenetic analysis was performed. The CaJAZ protein sequences were aligned using MAFFT v7.5.2 with default parameters. Subsequently, a maximum-likelihood (ML) tree was constructed using IQ-Tree v2.4.0 (Minh et al., 2020). The optimal protein substitution model was determined by ModelFinder v2.5 (Kalyaanamoorthy et al., 2017) based on the Bayesian Information Criterion (BIC). Branch support was assessed through the UltraFast bootstrap approximation (UFboot) with 1000 replicates (Minh et al., 2013) and the SH-aLRT test (Anisimova et al., 2011). To provide a more comprehensive evolutionary context and facilitate cross-species comparative analysis, an additional phylogenetic analysis was conducted, incorporating JAZ protein sequences from pepper, Arabidopsis, and rice. The resulting phylogenetic tree topology was visualized and annotated using Chiplot (https://www.chiplot.online/).

### 2.5 Collinearity and selective pressure analysis

Intra- and inter-species collinearity of *JAZ* genes was analyzed using MCScanX v1.0.0 (Wang et al., 2012). For cross-species comparisons, reciprocal BLASTP searches (E-value < 1e-10, number of BlastHits = 5) were performed between pepper, Arabidopsis, and rice to identify syntenic gene pairs. Intra-species collinearity specifically within pepper was assessed using the CA272 genome as the reference. All collinearity results were visualized using TBtools (Chen et al., 2023). To evaluate evolutionary selection pressure, pairwise Ka (non-synonymous) and Ks (synonymous) substitution rates were calculated for orthologous *CaJAZ* gene pairs across the 20 accessions using KaKs_Calculator v3.0 (Zhang, 2022) with the YN (Yang and Nielsen) model. Ridgeline plots illustrating the distribution of Ka/Ks values were subsequently generated using the R packages ggridges v0.5.7 (https://github.com/wilkelab/ggridges) (Wilke, 2025) and ggplot2 v4.0.1 (Wickham, 2016). Furthermore, a heatmap depicting the proportion of *CaJAZ* genes with Ka/Ks values greater than one (Ka/Ks > 1) was plotted using the R package ComplexHeatmap v2.26 (Gu, 2022).

### 2.6 Analysis of promoter sequences of JAZ family members

To evaluate the evolutionary conservation of cis-regulatory elements (CREs) in the promoter regions of *CaJAZ* genes across the 20 representative pepper genomes, the 2,000-bp sequences upstream of the start codon (ATG) for each *CaJAZ* member were extracted using TBtools v2.31 (Chen et al., 2023) and submitted to PlantCARE (http://bioinformatics.psb.ugent.be/webtools/plantcare/html/, accessed on 4 March 2026) for the prediction of their cis-regulatory element (CRE) distributions (Lescot et al., 2002). For each *CaJAZ* gene, the mean abundance of specific CREs and their conservation frequency (the proportion of accessions containing the specific motif) were calculated across all 20 accessions. The CREs were visualized to demonstrate the robust regulatory landscape of the JAZ family in peppers. Predicted elements were categorized by abiotic stress responsiveness, hormone-related, plant growth and development, and light responsiveness, and visualized as dot plots using the R package ggplot2 v4.0.1 (Wickham, 2016).

### 2.7 RNA−seq data analysis

*RNA−seq data:* The transcriptomic data of pepper leaves at the 6-true-leaf stage (0, 1, 3, 6, 12, and 24 h) under four key hormonal treatments (ABA, ET, MeJA, and SA) were obtained from https://www.ncbi.nlm.nih.gov/sra/ with the Sequence Read Archive accession number SRP265260 (Lee et al., 2020). We selected this dataset because it offers a systematic and high-quality temporal profiling of the pepper leaf transcriptome under precisely controlled hormonal treatments. This profiling is ideally suited for analyzing the dynamics of the JAZ family. The expression data employed are from high-depth RNA-seq (187.8 Gb of transcriptome data, 2.4 Gb per sample) with three biological replicates. For each replicate, leaves were gathered from four healthy plants, which ensures inherent quantitative accuracy (Lee et al., 2020).

*Quality Control and Pre-processing:* Raw sequencing reads were initially assessed for quality using FastQC v0.11 (https://www.bioinformatics.babraham.ac.uk/projects/fastqc/). Subsequently, low-quality reads and adapter sequences were meticulously removed using Trimmomatic v0.40 (Bolger et al., 2014) in paired-end mode. Specifically, leading and trailing low-quality bases (Phred score < 3), reads with an average quality score below 20 within a 4-base sliding window, and reads shorter than 36 bp after trimming were discarded. Adapter sequences were identified and removed using Trimmomatic’s built-in Illumina adapter library.

*Genome Indexing and Read Alignment:* For accurate read alignment, a genome index was first constructed for the pepper reference genome CA272 using Hisat2 v2.2.1 (Kim et al., 2019). Processed RNA-seq reads were then aligned to the indexed genome using Hisat2 with default parameters. This splice-aware aligner generated Sequence Alignment/Map (SAM) files, which were subsequently converted to their binary equivalent (BAM) for downstream analysis.

*Transcript Assembly and Expression Quantification:* Following alignment, transcripts were assembled from the sorted BAM files for each sample independently using StringTie v3.0 (Pertea et al., 2015). StringTie concurrently performed expression quantification, providing transcript abundance estimates in fragments per kilobase of exon per million mapped reads (FPKM) or TPM (Transcripts Per Million), which normalize for gene length and sequencing depth. These FPKM/TPM values were used for subsequent differential expression analysis.

*JAZ Gene Expression Analysis:* To systematically investigate the temporal expression dynamics of *JAZ* genes in response to various phytohormone treatments, a comprehensive analysis pipeline was implemented. Differential expression analysis was performed using the DESeq2 package (Love et al., 2014) based on raw counts, and the shrinkage of effect size was applied to control for dispersion. Instead of relying solely on TPM ratios, statistical significance (Log2FoldChange and P-adj) was rigorously determined using the DESeq2 Wald test. Comparisons were made between each phytohormone treatment and its corresponding mock control at each specific time point. The resulting Log2FC values, along with their associated significance (FDR < 0.05), were visualized in heatmaps to illustrate the induction or repression of individual genes. For absolute quantification and visualization, TPM (Transcripts Per Million) values were calculated. The TPM expression profiles of the 12 identified *CaJAZ* genes were initially extracted and averaged across biological replicates. Family-level transcriptional dynamics were assessed by calculating the total TPM sum of all *CaJAZ* members within each sample. These collective response intensities (mean total TPM ± SD) were visualized using line plots to provide an overarching perspective of the family’s integrated response. Furthermore, for individual gene assessments, bar plots depicting mean TPM (± SD) were generated. The statistical significance of temporal expression changes within each treatment group was further validated using Tukey’s Honestly Significant Difference (HSD) test (alpha = 0.05). Statistical analyses and visualizations were performed in the R v4.4 using the packages ggplot2 v4.0.1 (Wickham, 2016), ComplexHeatmap v2.26 (Gu, 2022), and agricolae v1.3-7 (https://cran.r-project.org/web/packages/agricolae/refman/agricolae.html).

## 3 Results

### 3.1 Pan-genome landscape reveals distinct PAV and CNV patterns in pepper JAZ family

**Figure 1:**
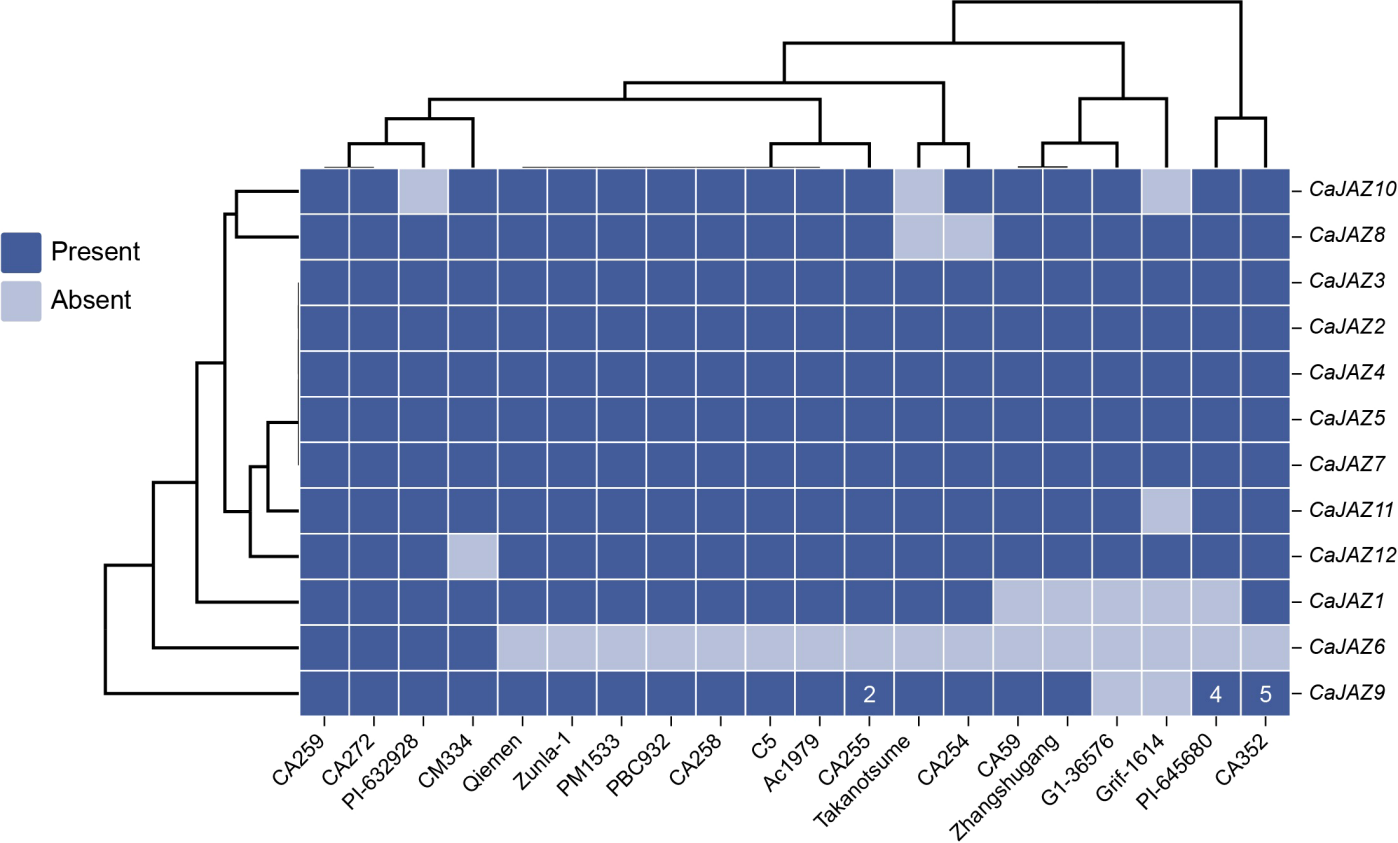
Pan-genome landscape of presence/absence variations (PAV) and copy number variations (CNV) of the *JAZ* gene family across 20 pepper accessions. The heatmap illustrates the distribution of 12 *CaJAZ* genes among 20 diverse pepper genomes. The phylogenetic tree at the top depicts the clustering of accessions based on their gene PAV patterns. A grey cell indicates the absence of the gene, while shades of blue indicate the presence, with the number inside the cell denoting the specific copy number (CNV).

To capture the full spectrum of genetic diversity within the pepper *JAZ* family, we performed a pan-genome analysis across 20 diverse accessions. Based on the presence/absence variation (PAV) heatmap, the 12 identified *CaJAZ* genes exhibited divergent evolutionary fates (**Figure 1**). A subset of genes, including *CaJAZ2*, *CaJAZ3*, *CaJAZ4*, *CaJAZ5*, and *CaJAZ7*, formed the highly conserved core gene set. These genes were present in all 20 re-sequenced genomes, underscoring their indispensable roles in pepper development. Additionally, *CaJAZ8*, *CaJAZ10*, *CaJAZ11*, and *CaJAZ12* were classified as near-core genes, being present in the majority of accessions but showing sporadic absences (e.g., *CaJAZ12* absent in Qiemen; *CaJAZ8* absent in CM334 and CA272).

Significant lineage-specific gene loss events were observed in specific subclades. The most striking variation was observed for *CaJAZ6*. While present in accessions such as CA259, CA272, PI-632926, and CM334, it was completely absent in a large cluster of genomes ranging from Qiemen to CA352, suggesting a major deletion event occurred during the diversification of these varieties. Similarly, *CaJAZ1* exhibited a cluster-specific absence in CA59, Zhangshugang, G1-36576, Grif-1614, and PI-645680. In contrast to gene loss, *CaJAZ9* demonstrated remarkable expansion via Copy Number Variation (CNV). While most accessions maintained a single copy, specific germplasms exhibited significant amplification. Notably, CA255 possessed 2 copies, PI-645680 contained 4 copies, and CA352 harbored 5 copies. This extensive CNV highlights *CaJAZ9* as an evolutionary “hotspot” subject to active duplication and potential neofunctionalization in specific germplasms.

### 3.2 Phylogenetic analysis and evolutionary relationships within *CaJAZs* family and between *JAZs* of model plants

**Figure 2:**
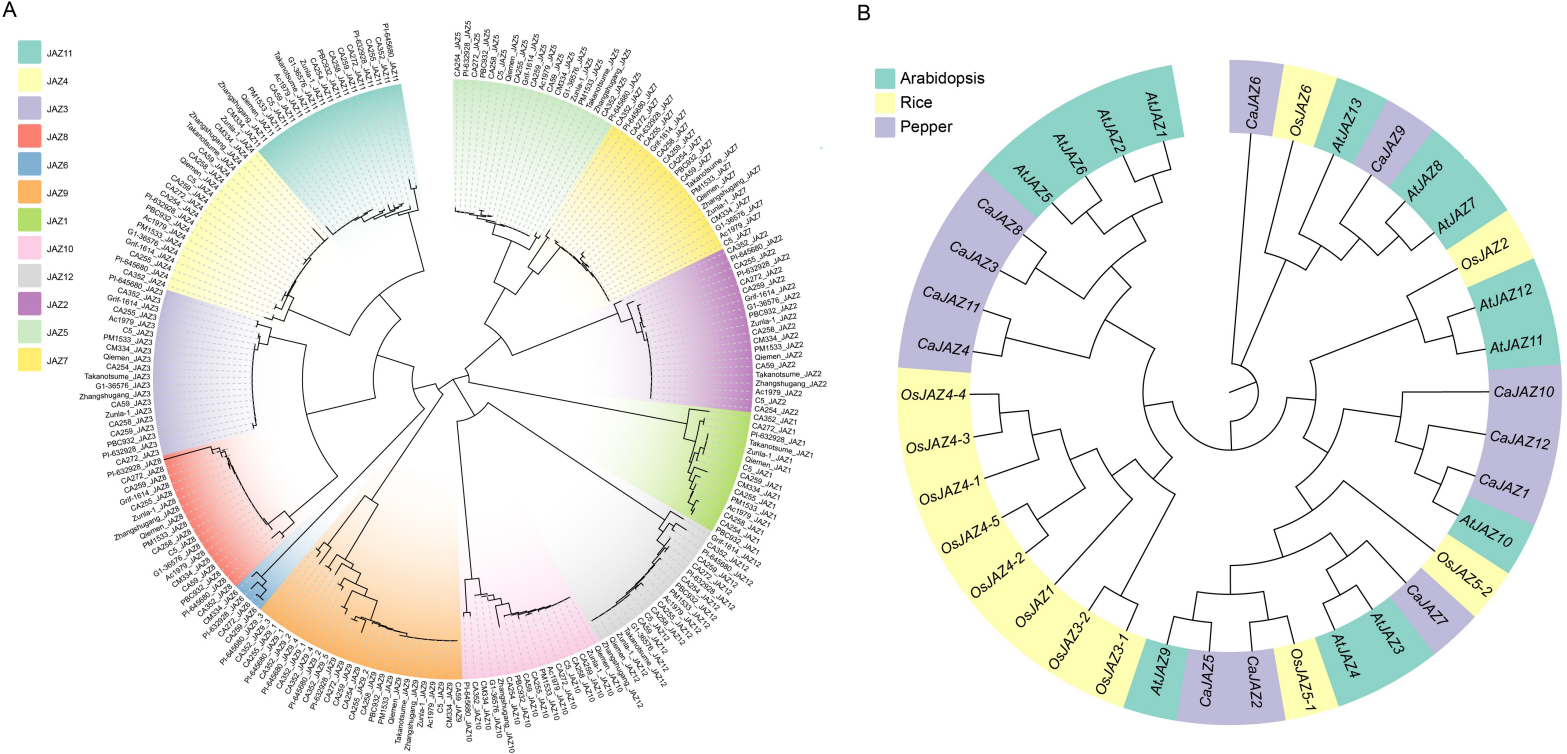
Phylogenetic relationships of *JAZ* proteins. **(A)** Phylogenetic tree of *JAZ* proteins across 20 pepper pan-genomes. An unrooted Neighbor-Joining (NJ) phylogenetic tree was constructed using full-length *JAZ* protein sequences identified from 20 pepper accessions. The tree resolves into 12 distinct clades, corresponding to the 12 *CaJAZ* members (CaJAZ1–CaJAZ12), which are distinguished by different background colors. **(B)** Comparative phylogenetic tree of *JAZ* proteins from Arabidopsis, rice, and pepper. Background colors indicate the species: teal for Arabidopsis, yellow for rice, and purple for pepper.

To elucidate the evolutionary relationships and divergence times of the JAZ family, we constructed a Maximum Likelihood (ML) phylogenetic tree using JAZ protein sequences from the 20 accessions (**Figure 2A**). The analysis resolved the pan-genome JAZ collection into 12 distinct, monophyletic clades. The majority of these clades, particularly those corresponding to the core genes (e.g., *CaJAZ2*, *CaJAZ4*, *CaJAZ7*), exhibited remarkably short branch lengths and tight clustering. This topology suggests that these JAZ members diverged prior to pepper speciation and have been maintained under strong purifying selection to preserve canonical functions.

Consistent with the PAV results, the *CaJAZ6* clade appeared notably sparse, visually confirming its dispensable nature in specific germplasms. In sharp contrast, the *CaJAZ9* clade displayed a complex topology indicative of recent expansion. Multiple paralogs from the same genome (e.g., five copies from CA352) clustered together within the *CaJAZ9* branch. This “species-specific clustering” pattern provides strong evidence that the expansion of *CaJAZ9* arose from recent tandem duplication events post-divergence, rather than ancient whole-genome duplication.

To clarify the evolutionary history and orthologous relationships of the JAZ family, we constructed a phylogenetic tree using JAZ protein sequences from pepper, Arabidopsis, and rice (**Figure 2B**). The phylogenetic analysis revealed that the JAZ proteins clustered into distinct subgroups, reflecting both conserved evolutionary history and lineage-specific divergence. As expected for dicotyledonous species, *CaJAZ* members generally showed closer phylogenetic affinity to *AtJAZs* than to *OsJAZs*. Most *CaJAZs* formed mixed clades with *AtJAZs* with high bootstrap support. For instance, *CaJAZ3*, *CaJAZ4*, *CaJAZ8*, and *CaJAZ11* clustered tightly with the *AtJAZ1/2/5/6* subgroup, suggesting they share a common ancestor that existed prior to the divergence of Solanaceae and Brassicaceae. Similarly, *CaJAZ7* was identified as the closest ortholog to the *AtJAZ3/4* pair, while *CaJAZ2* and *CaJAZ5* grouped with *AtJAZ9*.

The phylogenetic tree highlighted distinct gene expansion patterns between monocots and dicots. In the subgroup containing *AtJAZ10*, Arabidopsis possesses a single copy, whereas pepper contains three members (*CaJAZ1*, *CaJAZ10*, and *CaJAZ12*). This suggests a specific expansion event in the pepper lineage, potentially diversifying the function of the *JAZ10*-like repressors, which are known to be splice-variant prone and resistant to degradation. In contrast, a remarkable monocot-specific expansion was observed in the *OsJAZ4* clade (*OsJAZ4–1* to *OsJAZ4-5*), forming a distinct monophyletic branch with no direct pepper counterparts. This indicates that rice has undergone independent tandem duplications in this subfamily. *CaJAZ6* formed a relatively distinct clade with *OsJAZ6* and *AtJAZ13*/*CaJAZ9*, appearing more divergent from the major clusters. This distinct position, combined with its absence in several pepper accessions (as revealed by PAV analysis), suggests *CaJAZ6* might represent an ancient but less stable branch of the JAZ family.

### 3.3 Synteny and collinearity analysis of *CaJAZ* genes

To further elucidate the evolutionary origin and expansion mechanisms of the *CaJAZ* gene family, we performed comprehensive synteny analyses at the intra-species, pan-genome, and inter-species levels.

**Figure 3:**
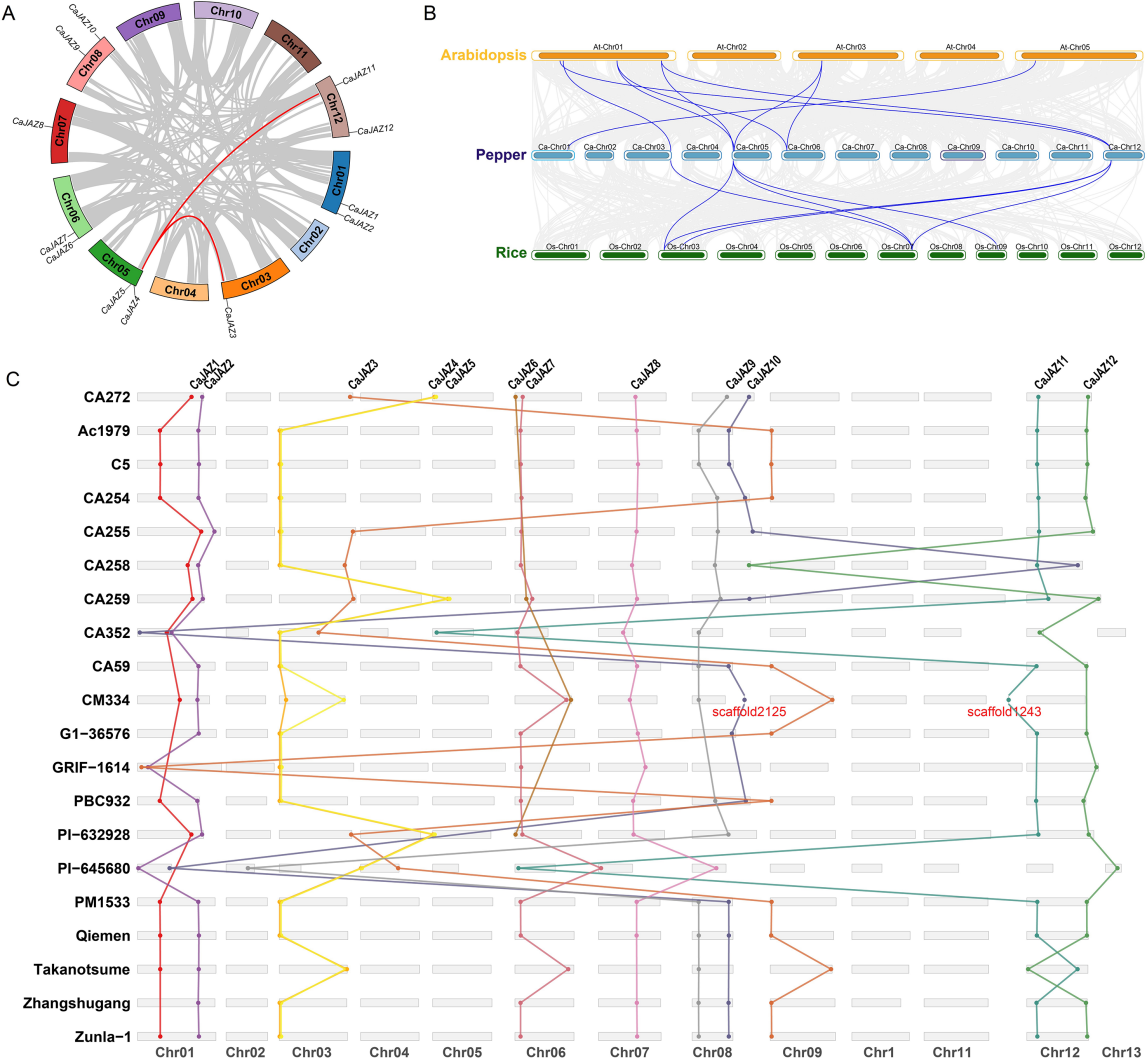
Synteny and collinearity analysis of the *JAZ* gene family. **(A)** Intra-species segmental duplication analysis within pepper. The circle plot visualizes the genomic positions of *CaJAZ* genes on the 12 chromosomes of pepper. Red curved lines connect duplicated gene pairs derived from segmental duplication events. **(B)** Inter-species comparative synteny analysis. Synteny blocks between pepper, Arabidopsis, and rice. Gray lines in the background represent collinear blocks across the entire genomes, while the highlighted blue lines connect orthologous *JAZ* gene pairs. **(C)** Pan-genome synteny analysis across 20 pepper accessions. The linear alignment displays the chromosomal locations and collinear relationships of *CaJAZ* genes among 20 diverse pepper genomes (using CA272 as a visual reference). The connecting lines indicate orthologous gene pairs.

1. *Intra-species Synteny and Gene Duplication Events.* Gene duplication is a primary driving force in the evolution of gene families. We analyzed the segmental duplication events of *CaJAZ* genes within the pepper genome CA272 (**Figure 3A**). The circle plot revealed that the *CaJAZ* family has undergone segmental duplication events driven by whole-genome duplication (WGD) or large segmental rearrangements. Specifically, distinct syntenic gene pairs were identified, highlighted by red connecting lines. Notably, *CaJAZ3* located on chromosome 03 exhibited collinear relationships with *CaJAZ4* on chromosome 5. *CaJAZ5* on chromosome 05 exhibited collinear relationships with *CaJAZ11* on chromosome 12. These results suggest that segmental duplication played a significant role in the expansion of the *CaJAZ* family.
2. *Stability of JAZ Loci Across the Pan-Genome.* To investigate the genomic stability of *CaJAZ* genes during pepper domestication and diversification, we constructed a linear synteny map across 20 pepper accessions using CA272 as the reference (**Figure 3B**). The analysis revealed a high degree of collinearity for all 12 *CaJAZ* genes. As shown in the figure, orthologous pairs (e.g., *JAZ1* to *JAZ1*, *JAZ2* to *JAZ2*) were connected by parallel lines with no significant chromosomal translocations or inversions observed in the *JAZ* gene loci across these diverse germplasms. This strict conservation of genomic context indicates that the *CaJAZ* genes are evolutionarily stable and functionally indispensable, consistent with their classification as core genes in our PAV analysis.
3. *Inter-species Comparative Synteny.* To trace the phylogenetic history of *CaJAZs*, we performed a comparative synteny analysis between pepper, Arabidopsis, and rice (**Figure 3C**). The syntenic relationships revealed a clear evolutionary hierarchy. We identified a substantial number of orthologous *JAZ* gene pairs between pepper and Arabidopsis, reflecting their closer divergence time within the Eudicot lineage. In contrast, fewer syntenic pairs were observed between pepper and rice. The higher density of syntenic blocks between pepper and Arabidopsis suggests that *JAZ* genes in Solanaceae share a more conserved ancestry and potentially similar functional roles with those in Brassicaceae compared to monocots.

### 3.4 Selection pressure analysis reveals adaptive evolution of specific *CaJAZ* members

**Figure 4:**
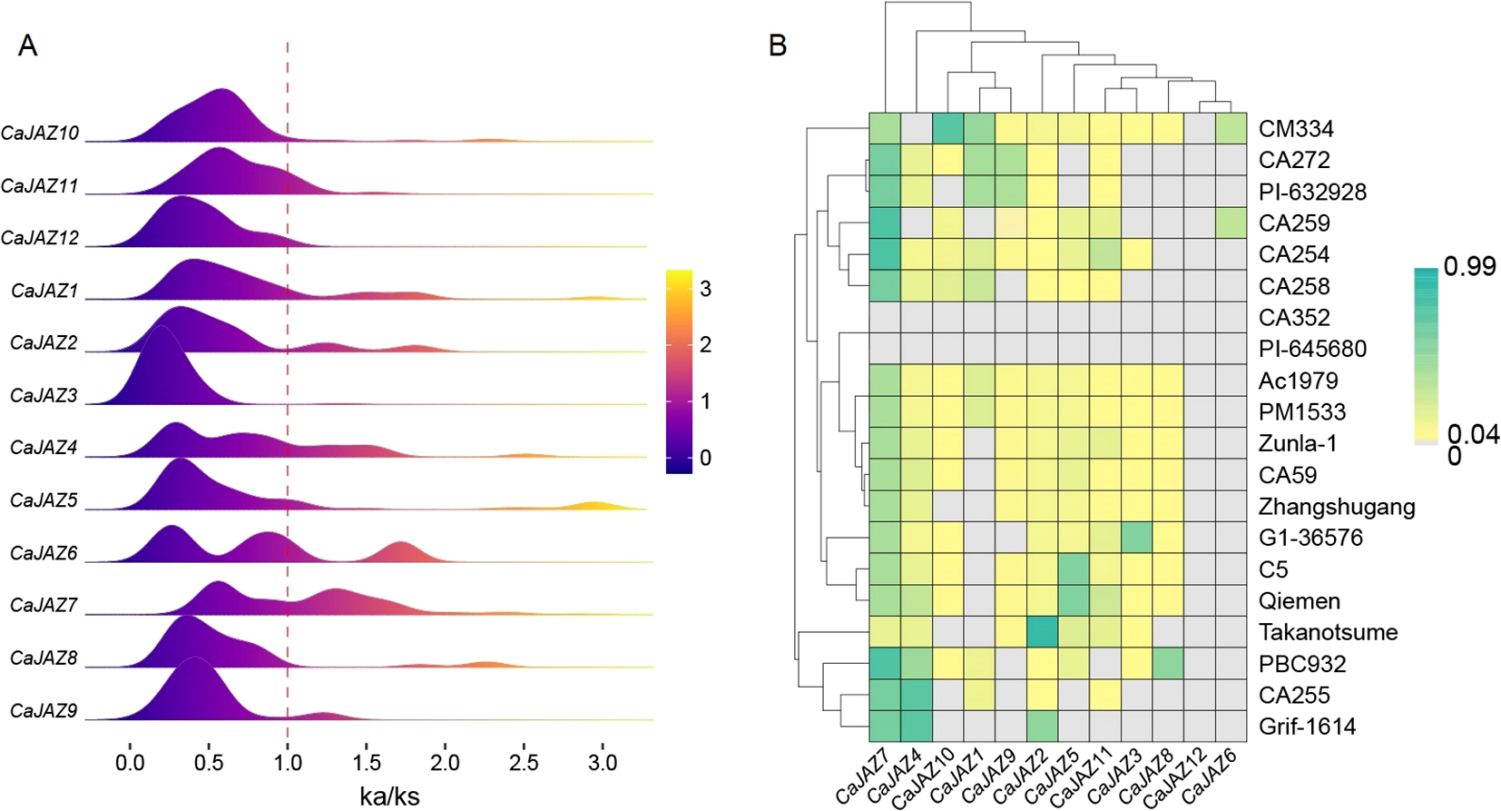
Evolutionary selection pressure on the *CaJAZ* gene family across 20 pepper genomes. **(A)** Ridge plot of Ka/Ks distributions. The plot shows the density distribution of Ka/Ks ratios for each *CaJAZ* gene across all homologous pairs identified in the pan-genome. The red dashed line marks the neutral selection threshold (Ka/Ks = 1). The purple peaks to the left indicate purifying selection, while tails extending to the right indicate instances of positive selection. **(B)** Heatmap of positive selection frequency. The heatmap displays the intensity of positive selection signals for each gene within each accession. The color scale represents the magnitude/frequency of Ka/Ks > 1, while grey indicates no positive selection detected or gene absence.

To understand the evolutionary forces driving the divergence of the *CaJAZ* family during pepper diversification and domestication, we calculated the non-synonymous to synonymous substitution ratios (Ka/Ks) for all *JAZ* orthologous pairs across 20 pepper genomes. The distribution of Ka/Ks ratios (**Figure 4A**) indicates that the *CaJAZ* family is predominantly under strong purifying selection. The majority of the distribution peaks are centered below 0.5 and strictly to the left of the neutral threshold. Genes such as *CaJAZ3*, *CaJAZ11*, and *CaJAZ12* exhibited extremely low Ka/Ks ratios, suggesting that amino acid substitutions in these proteins are largely deleterious and heavily constrained to maintain essential biological functions. However, signatures of positive selection were detected in specific members. Notably, *CaJAZ4*, *CaJAZ5*, *CaJAZ6*, and *CaJAZ7* displayed extended “tails” or secondary peaks crossing the Ka/Ks = 1.0 threshold. This distribution implies that while the gene family as a whole is conserved, specific *JAZ* members have undergone rapid adaptive evolution in certain lineages.

To further dissect these evolutionary signals across different germplasms, we constructed a heatmap showing the frequency and intensity of positive selection signals (Ka/Ks > 1) for each gene across the 20 accessions (**Figure 4B**). *CaJAZ4* and *CaJAZ7* stood out as evolutionary hotspots. As shown in the heatmap, these two genes exhibited high Ka/Ks signals across the majority of the accessions. This suggests that *CaJAZ4* and *CaJAZ7* are continuously evolving under diversifying selection pressure across the *Capsicum* genus. The hierarchical clustering of accessions revealed distinct evolutionary histories. A cluster including CA352 and PI-645680 showed almost no positive selection signals for most *JAZ* genes, indicating a highly conserved or ancestral state in these lineages. In contrast, accessions like CM334 exhibited widespread positive selection signals, reflecting dynamic adaptation in these varieties. Notably, *CaJAZ12* appeared as “grey” (value 0) in the heatmap across all accessions. Combined with our PAV analysis, this likely reflects the gene’s absence in these genomes rather than a lack of selection, further confirming its status as a dispensable gene.

### 3.5 Promoter Cis-element landscape highlights light and hormone signaling hubs

**Figure 5:**
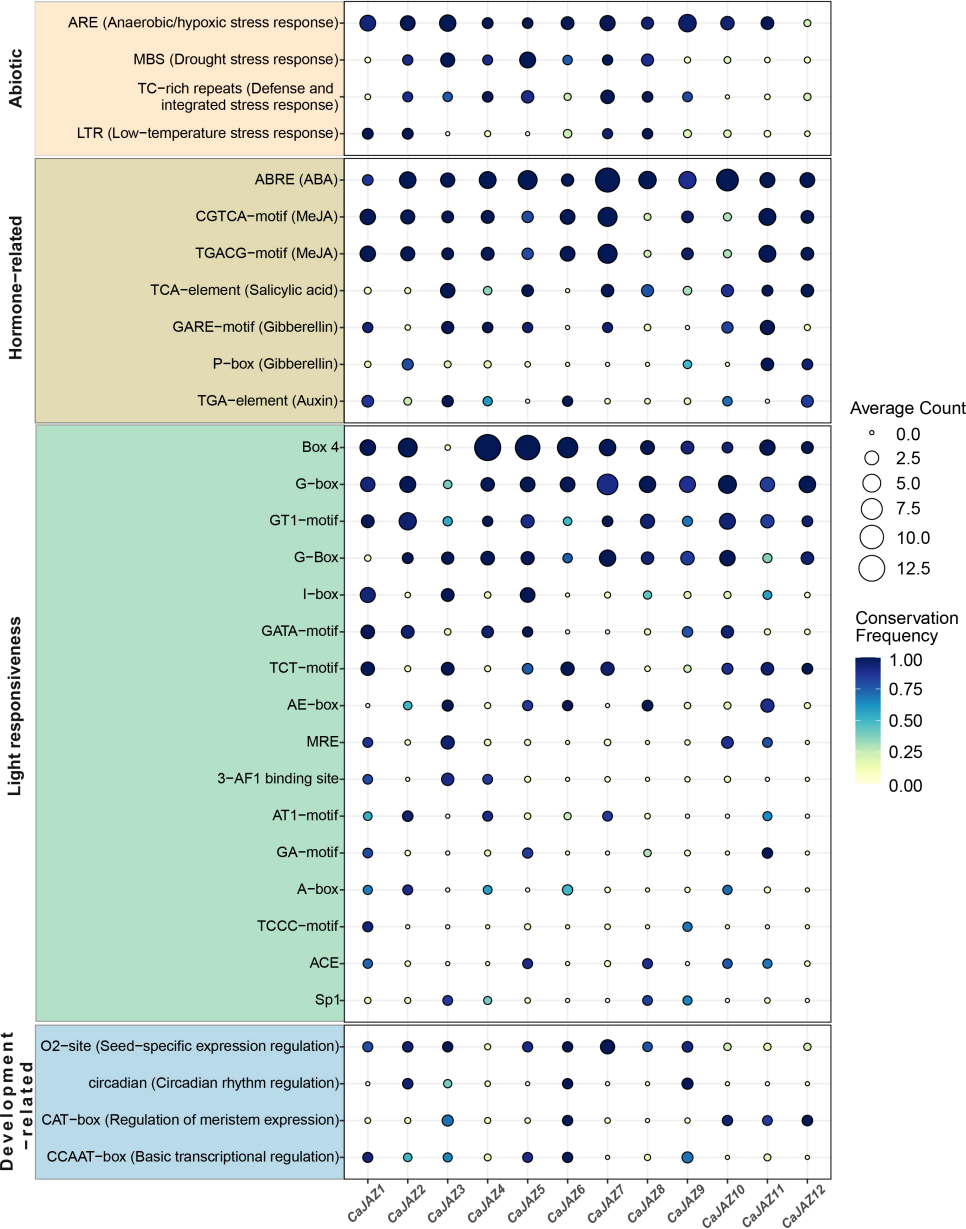
Conservation and distribution of the cis-regulatory elements (CREs) in *CaJAZ* gene promoters across 20 pepper accessions. Bubble plot showing the abundance and conservation of the CREs. The size of each bubble represents the mean count of the motif across 20 accessions, and the color gradient from light yellow to dark blue indicates the conservation frequency.

Promoter CREs play a pivotal role in regulating gene expression, particularly in response to environmental cues and during endogenous developmental processes. To elucidate the transcriptional regulatory mechanisms underlying *CaJAZ* genes, we performed a bioinformatics analysis of the promoter regions of all *CaJAZ* genes to identify enriched CREs. The analysis revealed a rich landscape of CREs within the *CaJAZ* gene promoters (**Figure 5**). A high diversity and significant enrichment of light-responsive CREs were observed within the *CaJAZ* gene promoters. Elements such as G-Box, Box 4, AE-box, and TCT-motif were commonly present, exhibiting high average counts and conservation across different *CaJAZ* genes. Notably, core light-responsive elements like G-Box (including its variant Box 4) and I-box showed high conservation and copy numbers in the promoters of almost all *CaJAZ* members. This widespread presence suggests that light is a crucial environmental factor regulating the expression of most *CaJAZ* genes. Such extensive involvement implies a significant role for the *CaJAZ* gene family in mediating light signal transduction pathways, potentially participating in physiological processes such as circadian rhythms, photomorphogenesis, and photosynthesis in pepper.

Beyond light-responsive elements, a substantial number of plant hormone-related CREs were identified in *CaJAZ* promoters, underscoring the potential involvement of JAZ proteins in hormonal signaling crosstalk. These hormone-responsive elements include ABRE (ABA), TGACG-motif and CGTCA-motif (MeJA), TCA-element (SA), TGA-element (auxin), and P-box and GARE-motif (gibberellin). Among these, ABRE, TGACG-motif, and CGTCA-motif displayed high conservation and relatively higher average counts in *CaJAZ* gene promoters, particularly in multiple *CaJAZ* members including *CaJAZ1*/*2*/*3*/*4*/*6*/*7*/*9*/*11*/*12*. This indicates that *CaJAZ* gene expression is finely regulated by various plant hormones, with a particularly strong association with ABA and MeJA signaling pathways. Given the central repressive role of JAZ proteins in the jasmonate signaling pathway, the enrichment of MeJA-responsive elements in their promoters further confirms a self-regulatory feedback mechanism for *JAZ* gene expression. Concurrently, the presence of other hormone-responsive elements suggests that the JAZ family coordinates through complex hormonal signaling networks during pepper growth, development, and stress responses.

In addition to light and hormone signals, *CaJAZ* gene promoters also contained CREs associated with abiotic stress responses (e.g., LTR, TC-rich repeats, MBS, ARE) and plant growth and development (e.g., CCAAT-box, CAT-box, circadian, O2-site). The discovery of these elements further broadens our understanding of the multifunctional nature of *CaJAZ* genes. For instance, the presence of MBS and LTR suggests that *CaJAZ* genes may play a role in pepper’s response to abiotic stresses such as drought and cold. The prevalence of circadian elements indicates that *CaJAZ* gene expression might be regulated by the circadian clock, thereby influencing periodic physiological activities of the plant.

### 3.6 Sequence diversity and structural architecture of the Jas motif across pepper pan-genomes

To elucidate the molecular basis of COI1-JAZ interactions in pepper, we systematically aligned the Jas motif (the primary degron responsible for JA-Ile perception) across the 12 CaJAZ subfamilies from the 20 assembled pepper genomes. The alignment revealed a highly stratified architectural divergence: absolute conservation at the hormone-anchoring site coupled with radical sequence variations at the receptor-interacting interfaces.

**Figure 6:**
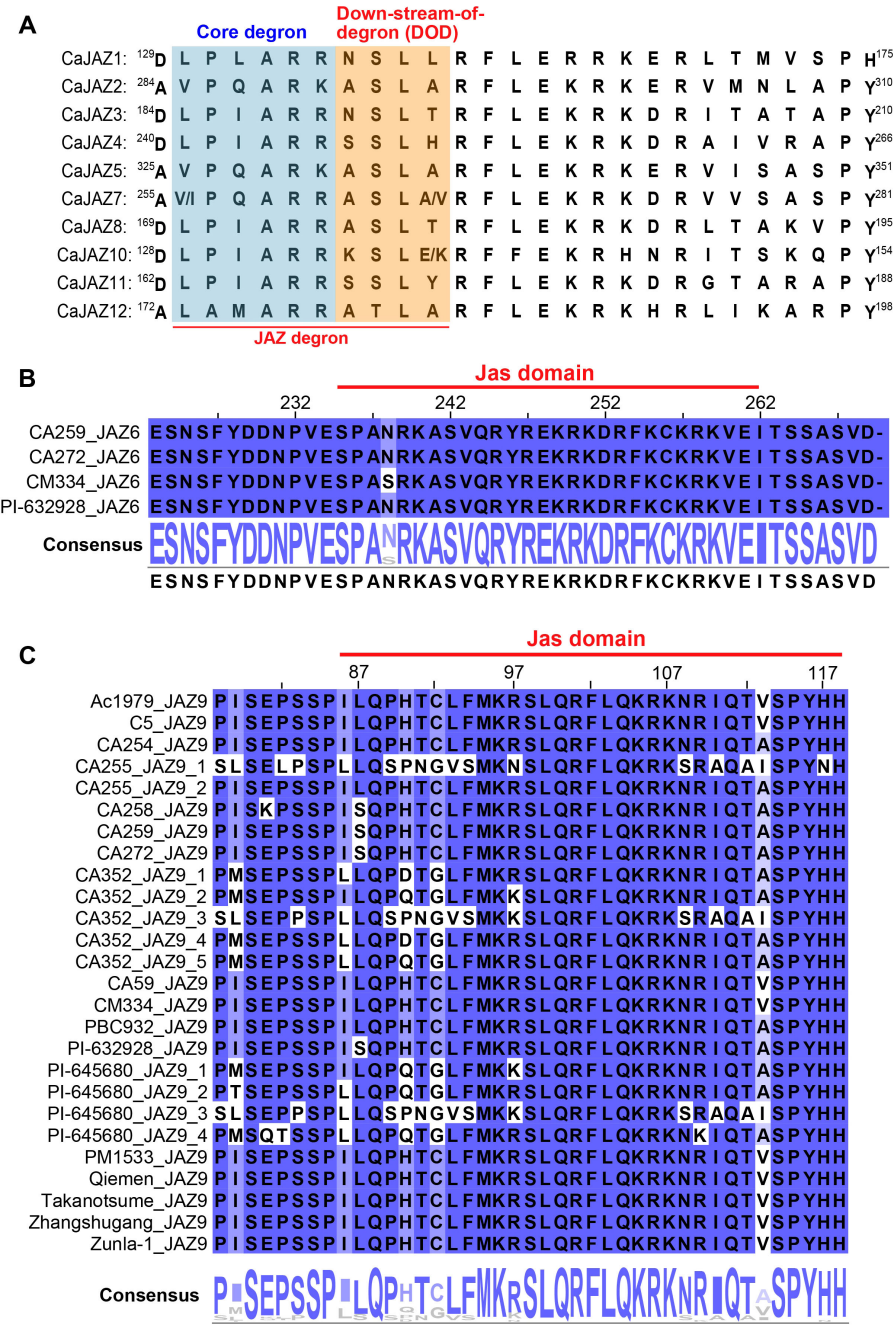
Structural diversity and sequence alignment of the Jas motif across the pepper JAZ pan-genome. **(A)** Multiple sequence alignment of the Jas motif from representative CaJAZ members (excluding CaJAZ6 and CaJAZ9). The bipartite “JAZ degron” responsible for COI1-receptor interaction is highlighted, comprising the N-terminal core degron (blue shading; displaying canonical LPIAR[R/K] or non-canonical variants such as VPQARK and Solanaceae-specific LAMARR) and the Downstream-of-Degron (DOD) anchor region (orange shading; strictly following the X-S-L-X pattern). The C-terminal amphipathic α-helix typically terminates with the highly conserved Pro-Tyr (P Y) structural anchor, with a notable natural anomaly in CaJAZ1 (P H). Intraspecific polymorphic sites are indicated by slashes (e.g., V/I or A/V). **(B)** Sequence alignment of the structurally attenuated CaJAZ6 clade across selected pepper accessions. The predicted Jas domain (indicated by the red line) harbors a highly polar mutated degron (PANRK/S) and exhibits a severe architectural truncation, completely lacking the universally indispensable C-terminal P-Y anchor. **(C)** Extensive sequence alignment of the expanded CaJAZ9 subclade across multiple pepper accessions, illustrating lineage-specific structural innovations. Key features include a novel [C/G]LFM-K[R/K] core degron, a strictly conserved S-L-Q DOD sequence, and a unique tandem Histidine extension (P-Y-H-H/N) at the extreme C-terminus. Notably, a naturally occurring putative loss-of-function allele is identified in CA255_JAZ9_1, which harbors a critical Lys-to-Asn (K–N) mutation at the essential hormone-anchoring site. In panels B and C, the intensity of the blue background indicates the degree of amino acid conservation, with the consensus sequence and sequence logo displayed below each alignment.

Firstly, the tandem basic amino acids R-R (Arg-Arg) or R-K (Arg-Lys) at the C-terminus of the core degron, which are indispensable for JA-Ile binding, were strictly conserved across almost all CaJAZ members. However, the N-terminal receptor-interacting region exhibited distinct evolutionary configurations. Approximately half of the members (CaJAZ3/4/8/10/11) possessed the canonical LPIARR/K motif, while CaJAZ12 harbored a Solanaceae-specific rare variant, LAMARR (**Figure 6A**). A non-canonical VPQARK/R degron was identified in CaJAZ2/5/7. The CaJAZ9 subclade exhibited extreme divergence, harboring a novel [C/G]LFM-KR/KK consensus where the N-terminal interface is substituted with bulky hydrophobic residues (**Figure 6B**). Strikingly, a naturally occurring loss-of-function allele was identified in accession CA255 (CA255_JAZ9_1), which harbors a severe K-N (Lys-Asn) mutation that eradicates the requisite basic charge for hormone anchoring.

Immediately downstream of the core degron, the Downstream-of-Degron (DOD, X-S-L-X) region displayed profound inter-clade polymorphism. CaJAZ2/5/7 shared an identical A-S-L-A DOD sequence, known for conferring high receptor affinity (**Figure 6A**). In contrast, other members exhibited diverse substitutions (e.g., NSLT, SSLH), with CaJAZ10 possessing a highly charged KSLE/K motif, and CaJAZ9 showing a universally conserved but sterically hindered S-L-Q sequence (**Figure 6B**).

Finally, while the C-terminal amphipathic α-helix and its terminal Pro-Tyr (P-Y) anchor were highly conserved in most members, three remarkable lineage-specific anomalies were discovered (**Figure 6A**). First, the terminal Tyrosine (Y) was substituted with Histidine (H) in CaJAZ1 (P-H), and extended to a tandem Histidine tail in all CaJAZ9 proteins (P-Y-H-H). Most strikingly, structural parsing of the CaJAZ6 clade revealed a severe architectural truncation (**Figure 6B**). Not only is its core degron highly polar (PANRK) and its DOD altered (A-S-V-Q), but CaJAZ6 completely lacks the universally conserved P-Y terminal anchor, precipitating immediately into a disorganized, highly charged tail (…KCKRKVE…).

### 3.7 Expression profiles of the *CaJAZ* gene family in response to phytohormone treatments

**Figure 7:**
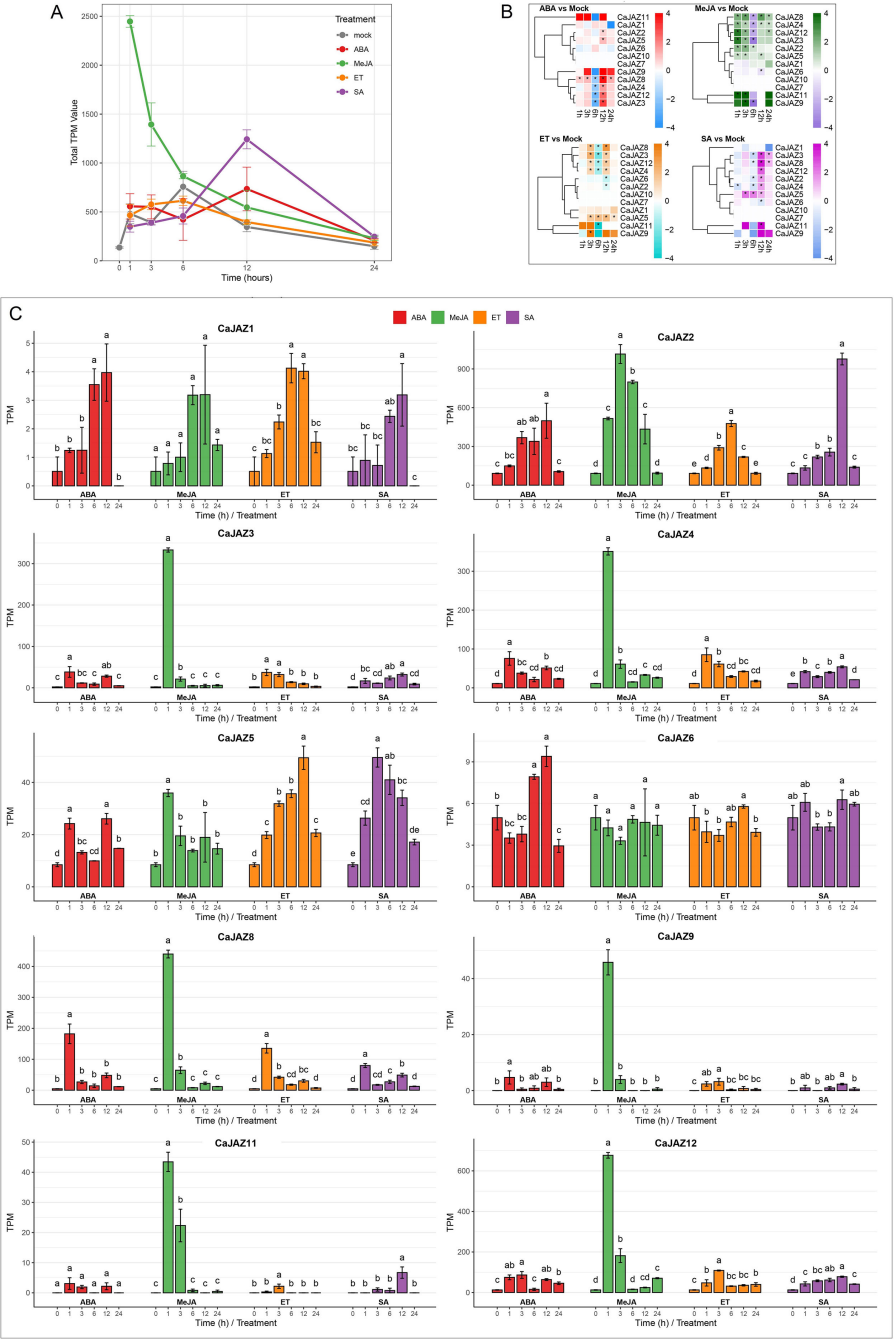
Spatiotemporal expression patterns of *CaJAZ* genes under hormonal treatments. **(A)** Family-level transcriptional dynamics. The line graph illustrates the temporal changes in total *CaJAZ* expression (calculated as the sum of TPM values for all 12 CaJAZ members) under Mock, ABA, MeJA, ET, and SA treatments over a 24-h period. This represents the collective response intensity of the entire JAZ family within the transcriptome pool. Values represent mean ± SD (n = 3). **(B)** Differential expression analysis of *CaJAZ* genes. Heatmaps display the Log2 Fold Change (Log2FC) of individual *CaJAZ* genes relative to their corresponding Mock controls at each time point. Log2FC values and statistical significance were rigorously determined using the DESeq2 Wald test to account for biological variation. Asterisks (*) indicate significant differential expression (adjusted P < 0.05). Hierarchical clustering reveals the relative sensitivity and specific induction patterns of individual genes independent of their basal expression levels. **(C)** Absolute expression profiles of individual *CaJAZ* genes. Bar charts show the absolute TPM values for 10 transcriptionally active *CaJAZ* genes (*CaJAZ7* and *CaJAZ10* were excluded due to undetectable expression levels). These plots reveal the actual transcript abundance and functional contribution of each gene to the total JAZ pool. Different letters (a–e) indicate statistically significant differences among time points within each treatment group (One-way ANOVA followed by Tukey’s HSD *post-hoc* test, P < 0.05). Values represent mean ± SD (n = 3).

To comprehensively characterize the transcriptional responses of the *CaJAZ* gene family to phytohormones, we analyzed both individual gene expression patterns (log_2_FC relative to mock) and family-wide transcript abundance (TPM) in pepper leaves treated with MeJA, ABA, ET, and SA over a 24-h time course (**Figures 7A–C**). At the family-wide level, MeJA elicited the most robust and acute transcriptional response. Total *CaJAZ* TPM reached a massive peak at 1 h post-MeJA treatment and then gradually decreased (**Figure 7A**), indicating a rapid but transient JA-driven activation of the repressor family. In contrast, ABA, ET, and SA induced broader, delayed, or less intense family-wide responses (e.g., SA peaking at 12 h).

Crucially, gene-specific expression profiling (**Figures 7B, C**) revealed striking intra-family divergence. Based on their transcriptional kinetics and hormonal crosstalk capabilities, the *CaJAZ* family can be broadly classified into three distinct transcriptional archetypes:

1. *Acute Early Responders (CaJAZ3/4/8/9/11/12)*: This group represents the canonical MeJA-driven feedback loop. These genes exhibited extremely low basal expression but underwent explosive transcriptional induction as early as 1 h under MeJA treatment. For example, the transcript abundance of *CaJAZ3* and *CaJAZ4* surged from near zero to >300 TPM at 1 h, followed by a precipitous drop toward baseline by 3–6 h (**Figure 7C**). *CaJAZ11* and *CaJAZ12* similarly peaked at 1 h and 3 h, respectively, indicating their primary roles as rapid “surge protectors” in jasmonate signaling.

2. *Broad-Spectrum Integrators (CaJAZ1/2/5)*: While retaining strong MeJA responsiveness at early time points, this subset uniquely exhibited significant transcriptional upregulation under diverse hormonal cues, suggesting roles in complex signaling crosstalk. Notably, *CaJAZ5* displayed substantial and prolonged induction under ET (peaking between 6–12 h) and SA (peaking at 12 h). *CaJAZ2* displayed an exceptionally strong late-stage response to SA, peaking at ~900 TPM at 12 h. Additionally, *CaJAZ1*/*/2*/*5* were markedly induced by ABA, contributing to the secondary family-wide peak observed at 12 h (**Figure 7A).**

3. *The Recalcitrant Non-Responder (CaJAZ6)*: Remarkably, *CaJAZ6* appeared completely decoupled from the MeJA-induced negative feedback loop. Unlike the acute early responders, its transcript levels remained constitutively low (TPM ~3–5) and showed no statistically significant peak across the entire MeJA time course (**Figure 7C**). Although it exhibited a mild, delayed response to ABA at 12 h, its profound “transcriptional deafness” to exogenous MeJA highlights its highly divergent regulatory mechanism within the pepper *JAZ* family.

## 4 Discussion

The *JAZ* gene family serves as the central “switch” in the Jasmonate signaling pathway, balancing the trade-off between plant growth and defense responses (Major et al., 2017). While extensive studies have been conducted in model plants like Arabidopsis and rice, the evolutionary dynamics and functional divergence of *JAZ* genes in pepper—a globally important crop—remain largely unexplored. Our pan-genome analysis provides the first comprehensive atlas of the *CaJAZ* family, revealing a complex landscape shaped by lineage-specific expansion, structural variation, and transcriptional specialization.

### 4.1 Evolutionary expansion: solanaceae-specific strategies

Our phylogenetic and synteny analyses reveal that the pepper *JAZ* family has undergone a distinct evolutionary trajectory compared to other major crop families. Unlike the massive expansion observed in Brassica crops (e.g., turnip and mustard), which is primarily driven by ancient whole-genome duplication (WGD) events (Cai et al., 2020; Jia et al., 2021), the expansion of the *CaJAZ* family appears to be driven by a combination of ancient segmental duplications and recent tandem duplications.

Specifically, the core genes (*CaJAZ2–5*/*7*/*11*) exhibit 1:1 orthology with tomato and eggplant *JAZs* (Sun et al., 2021; Li et al., 2024), suggesting they constitute the conserved “backbone” of the Solanaceae JA signaling network. However, the *CaJAZ9* subfamily represents a remarkable evolutionary hotspot. The identification of up to five tandemly duplicated copies of *CaJAZ9* in specific landraces (e.g., CA352) mirrors the expansion pattern seen in the *TaJAZ1* subfamily in wheat, which is linked to enhanced environmental adaptability (Zhai et al., 2024). We hypothesize that this gene dosage effect allows specific pepper varieties to mount a more robust or rapid transcriptional repression response under extreme stress conditions, or potentially provides genetic redundancy for neofunctionalization.

Furthermore, the reduced synteny between pepper and rice *OsJAZs* highlights the divergence between dicots and monocots. For instance, the monocot-specific expansion of the *OsJAZ4* clade (Shrestha and Huang, 2022) has no counterpart in pepper, indicating that lineage-specific duplications have tailored the JA pathway to adapt to distinct ecological niches—aquatic environments for rice versus tropical/subtropical terrestrial habitats for pepper.

### 4.2 The “Birth and Death” of *JAZ* genes: implications for domestication

The pan-genome PAV analysis uncovers a “birth-and-death” evolutionary dynamic that is invisible in single-reference genome studies. The widespread loss of *CaJAZ6* in a large cluster of accessions (including the reference Zunla-1) is particularly intriguing.

The widespread loss of *CaJAZ6* in specific lineages exemplifies adaptive gene loss during pepper diversification. Unlike the “energy economy” hypothesis that explains metabolic gene loss (Zhang et al., 2023), the dispensability of JAZ repressors likely reflects derepression benefits: loss-of-function mutations in negative regulators can enhance basal defense or stress tolerance without metabolic cost. Supporting this, SlJAZ25 in tomato functions as a susceptibility gene (S-gene), whose natural deletion enhances resistance to *Botrytis cinerea* (Sun et al., 2021). Similarly, *MsJAZ1* in alfalfa acts as a repressor of salt tolerance, with downregulation conferring stress adaptation (Cui et al., 2024). Thus, *CaJAZ6* deletion may represent a selected loss-of-susceptibility event, potentially releasing MYC2-mediated defense responses in specific ecological contexts. This “less-is-more” evolutionary strategy (Olson, 1999) appears particularly prevalent in the Solanaceae family, where *JAZ* gene redundancy allows for subfunctionalization and neofunctionalization through gene birth-and-death processes.

Conversely, the retention and expansion of *CaJAZ9* in other lineages suggest it confers a fitness advantage. This aligns with the “positive regulator” role observed for *StJAZ23* in potato, where overexpression promotes root growth and drought tolerance (Pu et al., 2025). Thus, the *CaJAZ* family evolution is a balance between purging deleterious repressors and amplifying beneficial regulators.

### 4.3 Adaptive evolution at the protein and promoter levels

Beyond copy number variation, adaptive signatures were detected at both protein sequence and promoter levels. The Ka/Ks analysis identified *CaJAZ4* and *CaJAZ7* as targets of strong positive selection. *CaJAZ7* is the ortholog of *AtJAZ9*, a key modulator of the tradeoff between necrotrophic and biotrophic pathogen resistance. Given that peppers are constantly challenged by diverse pathogens like *Phytophthora capsici* and viruses, the rapid evolution of *CaJAZ7* likely reflects an “evolutionary arms race,” where mutations in the JAZ protein interface allow it to evade pathogen effectors or interact with novel transcription factors (Howe and Yoshida, 2019).

The promoter architecture of the *CaJAZ* family in pepper reveals a complex regulatory landscape, where a high density of light-responsive and hormone-related CREs suggests these genes serve as molecular switches integrating environmental stimuli with endogenous signaling. The significant enrichment of G-Box and I-box motifs across *CaJAZ* promoters (e.g., *CaJAZ1*/*6*/*10*) points toward a robust linkage between light quality and jasmonate signaling. In Arabidopsis, light signaling directly modulates jasmonate sensitivity through interactions between PHYTOCHROME INTERACTING FACTORS (PIFs) and JAZ proteins (Chini et al., 2016). PIFs bind to G-box motifs to regulate target genes, while JAZ proteins physically inhibit PIF activity to suppress growth under specific conditions. The presence of these elements in *CaJAZ* promoters implies that pepper JAZ proteins are under the transcriptional control of light-induced factors, potentially mediating the “growth-defense trade-off” essential for survival (Yang et al., 2012).

The identification of ABRE and CGTCA-motif clusters underscores the central role of JAZ proteins in ABA and JA signaling crosstalk. JAZ proteins are established repressors of the JA pathway, degraded via the SCF-COI1 complex upon JA-Ile perception (Thines et al., 2007). Enrichment of MeJA-responsive motifs—particularly in *CaJAZ4*/*8*/*12*—suggests a negative feedback loop where JAZ proteins are upregulated to restore hormonal homeostasis (Zhao et al., 2024). Furthermore, the prevalence of ABREs indicates responsiveness to ABA, a key hormone for abiotic stress. JAZ proteins interact with components like MYC2, a master regulator of both JA and ABA responses (Lorenzo et al., 2004). This coexistence suggests *CaJAZ* genes function as signaling hubs, integrating drought or salt stress responses with JA-mediated defense (Liu et al., 2019).

The presence of MBS and LTR motifs indicates that the *CaJAZ* family extends beyond biotic defense into abiotic stress resilience. The diversity of these CREs highlights the potential for CaJAZ proteins to act as convergence points, allowing pepper to prioritize either development or defense through fine-tuned transcriptional responses to a fluctuating environment.

### 4.4 Structural diversification of the Jas motif provides a molecular basis for tuned JA responses in pepper

The structural integrity of the COI1-JAZ-JAIle tripartite complex relies heavily on a bipartite Jas motif: an N-terminal degron that captures the hormone as a “molecular glue,” and a C-terminal α-helix that acts as a “plug” over the binding pocket, locked in place by a terminal P-Y dipeptide (Thines et al., 2007; Sheard et al., 2010). Our pan-genomic analysis reveals that pepper has not merely conserved this mechanism but has dynamically uncoupled the core degron from the DOD and C-terminal anchors to evolve a highly stratified JAZ repressor matrix. Based on structural idiosyncrasies, we functionally categorize the CaJAZ repertoire into four interactive archetypes:

1. Despite possessing a non-canonical VPQARK degron, *CaJAZ2/5/7* uniformly harbor the A-S-L-A DOD sequence. Computational and empirical models in Solanaceae have demonstrated that the DOD sequence does not contact JA-Ile but forms peripheral anchor points with COI1; specifically, the ASLA motif confers the maximum binding affinity to the receptor (Saito et al., 2021). Thus, CaJAZ2, 5, and 7 likely function as primary responders, requiring minimal JA-Ile concentrations to trigger degradation for rapid defense priming.

2. *CaJAZ3/4/8/10/11/12*, which harbor the classic LPIAR(R/K) degron and are coupled with intermediate DOD variants, likely orchestrate standard jasmonate responses. The identification of the LAMARR core degron in CaJAZ12 is particularly noteworthy. This rare, proline-deficient motif was recently characterized in the other three Solanaceae species tomato (*Solanum lycopersicum*), potato (*Solanum tuberosum*), and the large white petunia (*Petunia axilaris*) (Garrido-Bigotes et al., 2019; Saito et al., 2021). Its absolute conservation in pepper CaJAZ12 solidifies it as a functional, Solanaceae-specific evolutionary innovation.

3. The unprecedented C-terminal modifications—the P-H tail in CaJAZ1 and the P-Y-H-H extension in CaJAZ9—introduce highly reactive imidazole rings directly adjacent to the receptor docking site. Given that Histidine is uniquely sensitive to physiological pH fluctuations (pKa ≈ 6.0), we postulate that these histidine-rich tails may act as allosteric pH sensors. They could potentially couple JAZ degradation rates to stress-induced cytoplasmic acidification (e.g., during PAMP-triggered immunity), independently of endogenous JA-Ile bursts. Furthermore, the discovery of the K-N mutation in CA255_JAZ9_1 suggests that specific pepper accessions harbor natural, non-degradable dominant repressors, highlighting ongoing intra-specific adaptive selection.

4. The most extreme evolutionary anomaly is CaJAZ6. The complete deletion of the indispensable P-Y anchor implies that the amphipathic α-helix cannot be stably tethered to the COI1 surface (Sheard et al., 2010). Without this hydrophobic “lock,” the structural plug over the JA-Ile pocket is thermodynamically prone to dissociation. We propose that CaJAZ6 functions as a highly recalcitrant, low-affinity repressor, serving as a critical molecular damper to prevent the hyper-activation of jasmonate responses and subsequent autoimmune growth stunting.

Crucially, the theoretical binding affinities predicted by this structural matrix are perfectly corroborated by our transcriptomic dynamics (discussed below), where canonical “sentinel” CaJAZs exhibited explosive transcriptional feedback, while the structurally compromised CaJAZ6 remained entirely unresponsive to exogenous MeJA.

### 4.5 Structural divergence of the Jas motif dictates transcriptional feedback dynamics and hormonal crosstalk

The JAZ proteins function as master repressors of JA signaling and pivotal nodes integrating diverse phytohormone pathways (Chini et al., 2007; Kazan and Manners, 2012). In the canonical JA signaling model, the rapid transcriptional induction of *JAZ* genes is not an upstream initiator, but a direct consequence of the rapid proteasomal degradation of pre-existing JAZ proteins. This degradation de-represses master transcription factors like MYC2, triggering a self-replenishing negative feedback loop to restore cellular homeostasis (Chung and Howe, 2009; Goossens et al., 2016). The integration of our pan-genomic structural predictions with transcriptomic profiles provides compelling evidence that the sequence diversification within the Capsicum JAZ family directly dictates their degradation kinetics and, consequently, their distinct transcriptional profiles.

As predicted by the structural model, members harboring the highly conserved LPIARR core degron (CaJAZ3/4/8/11) and the Solanaceae-specific variant LAMARR (CaJAZ12) exhibited a textbook “surge-and-decay” transcriptional profile. Their near-zero basal expression and explosive transcriptional induction at 1 h post-MeJA treatment perfectly align with their predicted capacity to form robust, high-affinity complexes with the COI1 receptor. These canonical members act as rapid “surge protectors”; their pre-existing proteins are swiftly degraded upon initial stress to launch defenses, and their transcripts are immediately replenished to prevent runaway immunity and minimize fitness costs.

Beyond basal JA signaling, CaJAZ genes serve as integration hubs for multiple phytohormone pathways. CaJAZ2 and CaJAZ5 represent a fascinating structural divergence, possessing the non-canonical VPQARK core combined with the ultra-high-affinity ASLA DOD region. Transcriptomically, they are not strictly MeJA-exclusive but serve as multi-hormone integrators, significantly responding to ET, ABA, and SA (e.g., the massive delayed peak of CaJAZ2 under SA treatment). The unique conformational stability conferred by the ASLA anchor might allow these specific JAZ proteins to interact promiscuously with diverse transcription factors (such as ABA-responsive or SA-responsive regulators) (Pauwels et al., 2010; Zhao et al., 2024). This structural adaptability buffers the antagonistic crosstalk typically observed between biotrophic (SA) and necrotrophic (JA/ET) defense pathways, enabling a finely tuned response to complex environmental challenges.

The most striking finding of our study is the profound “transcriptional deafness” of CaJAZ6 to MeJA treatment. While canonical CaJAZs exhibited massive spikes at 1 h, CaJAZ6 expression remained flat and constitutively low (**Figure 6C**). This transcriptomic anomaly elegantly corroborates our pan-genomic structural discovery: CaJAZ6 completely lacks the universally conserved Pro-Tyr (P-Y) C-terminal anchor and possesses a highly aberrant PANRK degron. Without the P-Y anchor to lock the Jas α-helix into the COI1 groove, the CaJAZ6 protein is predicted to be thermodynamically incapable of stable binding with the COI1-JA-Ile complex. Because the pre-existing CaJAZ6 protein cannot be efficiently degraded by MeJA, the MYC2-mediated feedback loop for this specific locus is never triggered, perfectly explaining its flat transcriptomic profile.

Collectively, this provides powerful multi-omics evidence that CaJAZ6 functions as a dominant, recalcitrant repressor. By escaping MeJA-induced degradation, CaJAZ6 acts as a constitutive “molecular brake,” ensuring that a basal level of growth-promoting transcription is maintained even during severe stress. Such “division of labor” within the pepper JAZ family reflects an evolutionary expansion designed to optimize the delicate trade-off between growth and defense (Wang et al., 2024).

## 5 Conclusion

This study constructed the first pan-genome atlas of the pepper JAZ family, revealing an evolutionary landscape shaped by strict conservation of core signaling modules and the dynamic expansion of variable members via tandem duplication. We demonstrate that lineage-specific variations, such as the adaptive loss of *CaJAZ6* and dosage amplification of *CaJAZ9*, likely drive adaptation to environmental stresses. A major scientific breakthrough of this study is the elucidation of a “structure-expression” paradigm. By mapping the structural divergence of the Jas motif—ranging from the high-affinity ASLA anchors in CaJAZ2/5 to the severe truncation of the P-Y tail in CaJAZ6—we provided compelling multi-omics evidence explaining how specific sequence anomalies dictate transcriptomic “deafness” and functional recalcitrance. Moreover, promoter architecture and transcriptomic profiling uncover sophisticated regulatory networks integrating light dependency and hormonal crosstalk. Collectively, these findings provide essential genetic resources for molecular breeding to fine-tune the growth-defense trade-off in climate-resilient peppers. Despite providing comprehensive pan-genomic insights, a limitation of this study is the reliance on transcriptomics as a molecular proxy, lacking *in vivo* experimental validation. Future research must prioritize empirical confirmation—including qRT-PCR, subcellular localization, and functional characterization via overexpression or CRISPR-Cas9 editing—to fully elucidate the specific biological roles of CaJAZ genes. Furthermore, associating specific structural anomalies and PAVs (e.g., the adaptive loss of CaJAZ6) with macroscopic field-level phenotypes, such as quantitative disease resistance, via GWAS remains a critical future direction.

## Data Availability

The original contributions presented in the study are included in the article/**Supplementary Material**. Further inquiries can be directed to the corresponding author.
